# Evaluating the Efficacy of Vagus Nerve Stimulation across ‘Minor’ and ‘Major’ Seizure Types: A Retrospective Analysis of Clinical Outcomes in Pharmacoresistant Epilepsy

**DOI:** 10.3390/jcm13144114

**Published:** 2024-07-14

**Authors:** Flavius Iuliu Urian, Corneliu Toader, Razvan-Adrian Covache Busuioc, Luca-Andrei Glavan, Antonio Daniel Corlatescu, Gabriel Iacob, Alexandru Vlad Ciurea

**Affiliations:** 1 Neurosurgical Department, University of Medicine and Pharmacy “Carol Davila”, 020021 Bucharest, Romania; flavius-iuliu.urian@drd.umfcd.ro (F.I.U.); razvan-adrian.covache-busuioc0720@stud.umfcd.ro (R.-A.C.B.); luca-andrei.glavan0720@stud.umfcd.ro (L.-A.G.); antonio.corlatescu0920@stud.umfcd.ro (A.D.C.); gabriel.iacob@umfcd.ro (G.I.); prof.avciurea@gmail.com (A.V.C.); 2 Neurosurgical Department, University Emergency Hospital Bucharest, 050098 Bucharest, Romania; 3 National Institute of Neurovascular Disease, 077160 Bucharest, Romania; 4 Sanador Clinical Center Hospital, 010991 Bucharest, Romania; 5 Medical Science Section, Romanian Academy, 050711 Bucharest, Romania

**Keywords:** pharmacoresistant epilepsy, vagus nerve stimulation (VNS), major seizures, minor seizures, seizure frequency reduction

## Abstract

**Background:** Evaluating the differential impact of vagus nerve stimulation (VNS) therapy across various seizure types, our study explores its efficacy specifically in patients with categorized minor and major seizures. **Methods:** We conducted a retrospective cohort study involving 76 patients with pharmacoresistant epilepsy treated at the University Emergency Hospital of Bucharest between 2021 and 2024. Seizures were classified as ‘minor’ (including focal-aware and non-motor/absence seizures) and ‘major’ (including focal to bilateral tonic-clonic and generalized motor seizures), based on modified International League Against Epilepsy (ILAE) criteria. This classification allowed us to assess the response to VNS therapy, defined by a 50% or greater reduction in seizure frequency at the 12-month follow-up. **Results:** Our findings reveal that major seizures respond more favorably to VNS therapy, significantly reducing both frequency and intensity. In contrast, minor seizures showed a less pronounced response in frequency reduction but noted improvements in neurocognitive functions, suggesting a nuanced benefit of VNS in these cases. **Conclusion:** The study underscores the importance of seizure type in determining the efficacy of VNS therapy, advocating for personalized treatment approaches based on seizure classification. This approach could potentially enhance clinical outcomes by tailoring VNS settings to specific seizure types, improving overall management strategies in pharmacoresistant epilepsy.

## 1. Introduction

The response among the various seizure types towards VNS for DRE varies greatly, thereby offering a broad canvas of inquiry [1,2]. Evidence indicates the nuanced nature of VNS outcomes for minor seizures, such as auras and focal-aware seizures [3,4]. Some reports state a decrease in the frequency of episodes with less intensity, while findings of other studies indicate a marginal decrease in seizure frequency but an increase in the frequency and amplitude of cognitive and behavioral functions related to it [1,5,6]. These data suggest that the effects of VNS include both seizure reduction and changes in neuropsychological quality of life [6,7,8]. In contrast, the response to major seizures is more demonstrable because the reports suggest a considerable decrease in the frequency and intensity of major seizures, such as tonic-clonic and myoclonic seizures, which are generally more difficult to deal with [3,4,9]. These conclusions are further supported by other studies suggesting more effective prevention of intense epileptic attacks with higher settings of stimulation [10,11,12]. Moreover, the parameterization of VNS therapy, such as pulse width and frequency modulation, the intensity of stimulation, etc., is a decisive factor regarding the therapy’s effectiveness [12,13].

## 2. Materials and Methods

### 2.1. Study Design

We conducted a retrospective cohort study involving 76 patients diagnosed with pharmacoresistant epilepsy. These individuals were evaluated and treated at the Epileptology Center and Neurosurgery II department at the University Emergency Hospital of Bucharest (SUUB) between 2021 and 2024. Participants were selected based on their unresponsive nature to conventional pharmacotherapy and subsequently treated with vagus nerve stimulation (VNS).

These patients underwent vagus nerve stimulation (VNS) therapy and had their seizures classified as either ‘minor’ (focal-aware and non-motor/absence seizures) or ‘major’ (focal to bilateral tonic-clonic and generalized motor seizures) according to modified International League Against Epilepsy (ILAE) criteria.

### 2.2. Demographics

The study population comprised 76 individuals aged between 20 and 65 years with drug-resistant epilepsy. The highest frequency was recorded within the range of 30–40 years, as shown below:

Age distribution: 19–29 years, 12 patients (15.79%); 30–40 years, 35 patients (46.05%); over 40 years, 29 patients (38.16%).

**Figure 1:**
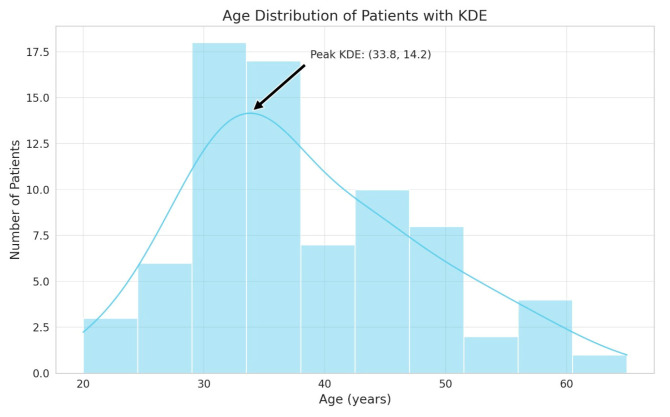
Age distribution graph.

Gender: There were 59.21% females, specifically 45 patients, while there were 40.79% males, specifically 31 patients, with the median age for females being 41 years and 32 years for males (Figure 1).

Regarding the distribution of sexes in our cohort, it consists of 59.2% females and 40.8% males. The median age of the participants is 36, with an age distribution from 20 to 65 years.

### 2.3. Seizure Categorization

In contrast to the standard International League Against Epilepsy (ILAE) seizure classification, our study uniquely adapted the classification criteria to categorize seizures into ‘minor’ and ‘major’ groups. This adaptation was imperative for evaluating the differential response to VNS therapy.

Minor seizures encompassed focal-aware seizures, non-motor/absence seizures, and all types of auras. These seizures were characterized by their non-disruptive nature to consciousness and limited motor presentation. Auras, as subjective sensory or psychic phenomena, were also included in this category due to their comparably milder clinical manifestation.

Major seizures included seizures that resulted in a significant alteration of consciousness, such as focal to bilateral tonic-clonic seizures and all forms of generalized motor seizures. The marked impairment and extensive motor involvement of these seizures signified a more severe epileptic event.

### 2.4. VNS Therapy and Evaluation Protocol

Patients underwent VNS therapy according to a standardized protocol. Initial post-implantation evaluations were scheduled at two-week intervals, during which stimulation parameters were incrementally adjusted to optimize the therapeutic balance between efficacy and tolerability. Recognizing the variability in follow-up adherence and individualized parameter adjustments, a comprehensive retrospective analysis was conducted at the 12-month mark. This timeframe was selected as it is considered sufficient for the stabilization of stimulation parameters tailored to each patient and is indicative of the long-term response to VNS therapy. The primary outcome measured was the reduction in the frequency of minor and major seizure types, with a favorable response defined as a 50% or greater reduction in seizure frequency.

### 2.5. Statistics

The predominant measurement analyzed was the proportion of patients who underwent a ≥50% reduction in seizure frequency subsequent to therapy with VNS. The threshold level was set as the optimum response level to this treatment. On the other hand, any reduction in seizure frequency that was below 50% fell under the not-so-good response category. This dichotomization clearly divided the patients into the responder and non-responder groups and helped in the easy assessment of the efficacy of VNS in different types of minor, major, and combined seizures. Regarding the statistical methods, descriptive statistics were used to summarize the baseline characteristics of the patient cohort. The primary outcome measure was the reduction in seizure frequency, defined as a 50% or greater reduction at the 12-month follow-up. Secondary outcomes included changes in seizure intensity and improvements in neurocognitive functions. Comparative analyses were conducted between the ‘minor’ and ‘major’ seizure groups to evaluate differential responses to VNS therapy. Statistical significance was determined using appropriate tests, such as chi-square tests for categorical variables and t-tests or Mann–Whitney U tests for continuous variables. A multivariate logistic regression analysis was performed to identify predictors of response to VNS therapy, adjusting for potential confounders. Results were reported with corresponding confidence intervals and *p*-values to indicate the robustness of the findings.

### 2.6. Response Categorization

- Positive response is defined as those patients who had >50% reduction in seizure frequency irrespective of minor, major, or combined seizure types.
- Non-responders are defined as patients who did not achieve at least a 50% reduction in seizure frequency.

We conducted a chi-square test of categorical data in order to compare the proportions of favorable and unfavorable responses between types of seizures. We tested differences between the statistically significant response rates of the groups in order to provide further insight into which types of seizures were most likely to respond to VNS.

In addition, we employed a logistic regression analysis to further understand the impact of seizure type on VNS treatment outcomes.

### 2.7. Ethics

All participants in the clinical study, under the agreed guidelines, agreed with informed consent specified by informed consent forms F-200-17 and F-562-21.

## 3. Results

### 3.1. Descriptive Statistics

**Figure 2:**
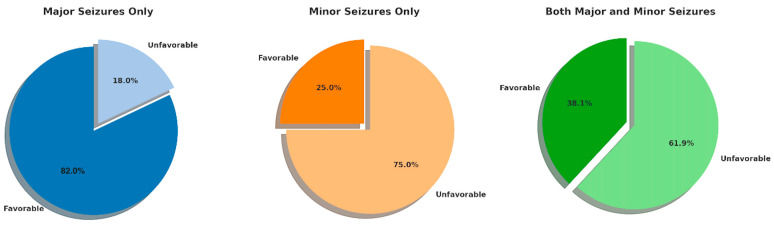
Distribution of clinical responses to VNS therapy by seizure type. A favorable response is defined as a reduction in seizure frequency of more than 50% after 12 months of VNS therapy, while an unfavorable response refers to a reduction of less than 50% in the same period.

Our cohort consisted of 76 patients. A very high number experienced major seizures (78.95%), whereas a significantly lesser, yet still substantial, number presented with minor seizures (48.68%). Intriguingly, a significant number of patients experienced both types of seizures (27.63%). Thus, the types of seizures in our study population are complicated and variegated in their clinical presentation. The clinical response to VNS therapy (Figure 2) was significantly varied between the different types of seizures:

- In minor seizures, a predominantly negative response was observed, where 75% did not experience the desired therapeutic effect. The favorable response rate in this subgroup was 25%, which reflects the low efficacy of VNS in this subpopulation.
- Major seizures only showed better efficacy of VNS, where 82.05% favored it; in distinct contrast, 17.95% did not respond favorably.
- This mixed response was seen with both major and minor seizures: 61.90% unfavorable and 38.10% favorable; hence, the presence of both types of seizures might complicate the therapeutic outcome.

### 3.2. Chi-Square Tests

The results of the chi-square test revealed a statistically significant association, with a chi-square value of 19.82 and a *p*-value of 0.00005. This significant result indicates that the type of seizures—whether major, minor, or both—affects the likelihood of achieving a favorable clinical response to VNS therapy (Table 1).

The chi-square value of 7.37, with a *p*-value of 0.0066, suggests a statistically significant association between having major seizures and a clinical response, indicating that patients with only major seizures are likely to exhibit different response patterns compared to others. Conversely, with a chi-square value of 17.20 and a *p*-value of 0.000034, there is a strong statistical significance, suggesting that minor seizures alone are associated with distinct clinical outcomes, specifically less favorable responses.

Both major and minor seizures revealed a chi-square value of 3.61, with a *p*-value of 0.0574, indicating that this category does not show a statistically significant difference from other groups. This suggests that the combined effect of having both types of seizures does not distinctly alter the clinical response patterns in a statistically significant way compared to other groups.

### 3.3. Logistic Regression Model

For the base category, whose patients suffer only minor seizures, the model gives us a coefficient of −1.0986; its *p*-value is 0.057. This would suggest that for this category, even though the response to treatment is not statistically significant, it does touch on relevance since it suggests that an underlying trend may exist in which patients suffering minor seizures might not react to the VNS therapy as much as those with major seizures. A far more positive reaction to VNS therapy occurs for the group suffering only from major seizures. The coefficient for this category is much higher than the others at 2.6184 and has a *p*-value of less than 0.001. Results thus strongly indicate that the patients with only major seizures improved much more, suggesting that VNS therapy is much more effective for patients suffering from more severe seizure manifestations.

For patients with major and minor seizures, the model gives a coefficient of 0.6131, and the associated *p*-value for it is 0.402. The interpretation is that the presence of both types of seizures does not significantly alter the chances for the best response compared to patients with the presence of only minor seizures. This seems to indicate a rather complex interplay between the two different kinds of seizures. As can be seen in Table 2, in which case the combined effects may neutralize or dilute the individual impacts seen in the isolation of major or minor seizures.

The model fit statistics—the pseudo-R-squared and very significant *likelihood ratio test* (LLR), at a *p*-value of 0.00003—all point to the high efficacy of the logistic regression approach in distinguishing among the responses of the different seizure type groups to VNS therapy.

## 4. Discussion

Treatment of the different types of seizures in drug-resistant epilepsy by VNS varies greatly, as demonstrated by studies focusing on the minor and major seizure categories.

### 4.1. Minor Seizures

A study by Müller et al. (2010) from Budapest had mixed results, with some efficacy found among nonfocal patients with epilepsy and generalized tonic-clonic seizures being the most responsive [14]. According to Danielsson et al. (2008), VNS was not clinically significant and had a positive impact on reducing the incidence of seizures in children with autism, meaning that such therapy has little or no effect in this population [1]. High-frequency VNS also evidenced a marked reduction in minor seizures in a meta-analysis of five RCTs with 439 participants carried out by Panebianco et al. (2015), in which the pooled RR was 1.73 (95% CI: 1.13 to 2.64) [5]. By contrast, Privitera et al. (2002) found in their systematic review that high-level VNS was more effective than low-level stimulation, with an odds ratio (OR) for a 50% or greater reduction in seizure frequency of 1.93 (95% CI: 1.1 to 3.3) [15].

### 4.2. Major Seizures

A meta-analysis made by Hajtovic et al. in 2022 found that vagal nerve stimulation was more effective for a number of major seizures, such as tonic-clonic and clonic, where it showed a reduction in drug-resistant epilepsy patients, the majority being tonic-clonic, with a decrease in the frequency of seizures [12]. Toffa et al. (2020) reported that VNS led to a 50–100% seizure frequency reduction in approximately 45–65% of patients with both minor and major seizures [16]. A population of 470 children and 113 adults with partial or generalized epilepsy, including major seizures such as LG syndrome, was studied for long-term outcomes of VNS by Morris et al. (2013), with a greater than 50% reduction in seizure frequency in 55% of these patients (95% CI: 50–59%), and the efficacy of the treatment increased over time [17].

## 5. Conclusions

The study’s findings demonstrated that patients with major seizures, such as focal to bilateral tonic-clonic seizures and generalized motor seizures, experienced a notably higher efficacy of VNS. The response rate was 82.05% for this subgroup, which was notably high compared with the others. This means VNS would be quite effective in epileptic episodes that could be very severe, whereby the depths of the seizure’s impacts on neurological functions are profound. The robust response in patients with major seizures underlines the potential of VNS as a critical intervention for the most adverse effects of DRE.

In contrast, patients with less intense seizures, focal-aware seizures, non-motor/absence seizures, and auras had a less dramatic response to VNS therapy, with a favorable response noted in only 25%; the majority of this group showed minimal change in seizure frequency.

An analysis of the results also revealed that in patients having both major and minor seizures, the results were mixed, and 38.10% responded positively. This fact only goes on to prove that patients suffering from both types of seizures may need an individualized approach to management. The coexistence of both types of seizures complicates the therapeutic approach further since the interaction between different seizure expressions might eventually influence the overall efficacy of VNS. 

## Figures and Tables

**Table 1:** Chi-square test results by seizure category.

Category	Chi-Square Value	*p*-Value
Major Seizures Only	7.368122633	0.006639
Minor Seizures Only	17.19507182	0.000034
Both Major and Minor Seizures	3.611757182	0.057372

**Table 2:** Logistic regression analysis of clinical response to VNS therapy by seizure type.

Variable	Coefficient	Standard Error	*z*-Value	*p*-Value	95% CI Lower	95% CI Upper
Minor Seizures	−1.09861229	0.577350269	−1.90285	0.05706	−2.230198023	0.032973445
Major Seizures	2.618438042	0.712348564	3.675782	0.000237	1.222260513	4.014615572
Combined	0.613104473	0.731612199	0.838018	0.40202	−0.820829089	2.047038034

## Data Availability

All data is available on reasonable request to the corresponding author.

## Abbreviations

CI = confidence interval; DRE = drug-resistant epilepsy; GTCS = generalized tonic-clonic seizures; ILAE = International League Against Epilepsy; LG = Lennox–Gastaut syndrome; LLR = likelihood ratio test; OR = odds ratio; RR = risk ratio; SUUB = University Emergency Hospital of Bucharest; VNS = vagal nerve stimulation.
